# Establishment and characterization of pygmy killer whale (*Feresa attenuata*) dermal fibroblast cell line

**DOI:** 10.1371/journal.pone.0195128

**Published:** 2018-03-29

**Authors:** Sun Yajing, Imran Rashid Rajput, Huang Ying, Yu Fei, Edmond Sanganyado, Li Ping, Wang Jingzhen, Liu Wenhua

**Affiliations:** 1 Marine Biology Institute, College of Science Shantou University, Shantou, Guangdong, P.R. China; 2 Faculty of Veterinary and Animal Sciences, Lasbela University of Agriculture, Water and Marine Sciences, Uthal, Balochistan, Pakistan; 3 Ocean College, Qinzhou University, Qinzhou, Guangxi, P.R. China; University of Rochester School of Medicine and Dentistry, UNITED STATES

## Abstract

The pygmy killer whale (*Feresa attenuata*) (PKW) is a tropical and subtropical marine mammal commonly found in the Atlantic, Indian and Pacific oceans. Since the PKWs live in offshore protected territories, they are rarely seen onshore. Hence, PKW are one of the most poorly understood oceanic species of odontocetes. The dermal tissue comes primarily from stranding events that occur along the coast of the Shantou, Guangdong, China. The sampled tissues were immediately processed and attached on collagen-coated 6-well tissue culture plate. The complete medium (DMEM and Ham’s F12, fetal bovine serum, antibiotic and essential amino acids) was added to the culture plates. The primary culture (PKW-LWH) cells were verified as fibroblast by vimentin and karyotype analyses, which revealed 42 autosomes and two sex chromosomes X and Y. Following transfection of PKW-LWH cells with a plasmid encoding, the SV40 large T-antigens and the transfected cells were isolated and expanded. Using RT-PCR, western blot, immunofluorescence analysis and SV40 large T-antigen stability was confirmed. The cell proliferation rate of the fibroblast cells, PKW-LWHT was faster than the primary cells PKW-LWH with the doubling time 68.9h and 14.4h, respectively. In this study, we established PKW dermal fibroblast cell line for the first time, providing a unique opportunity for *in vitro* studies on the effects of environmental pollutants and pathogens that could be determined in PKW and/or Cetaceans.

## Introduction

Pygmy killer whales (*Feresa attenuata*) are tropical and subtropical delphinid with extremely aggressive behaviour, they are rarely seen onshore and are one of the most poorly known oceanic species of odontocetes [1]. PKW habitats in deep and warm waters beyond the onshore continent, and very rarely seen close to the edges of the continental shelf (except near few island, where the water is very deep). [2]. Current population estimation of PKW by IUCN, approximately 38,900, is for the eastern tropical Pacific population. [3], whereas in the eastern tropical Pacific, PKW were ranked as 12^th^ out of the 13 species which exists [4]. Current studies primarily focused on PKW sightings [2], that is a visual survey of PKW population estimation, regions, occasional strandings and movements [5]. In recent years, advancement in PKW research has extended to assess satellite movement by tagging [6]. However, threats of overfishing, water pollution, and heavy marine traffic are rapidly threatening the population of marine mammals. While, recent estimates revealed declining populations which may accelerate in the future, thus threatening PKW with extinction [7]. Extinction is known as the permanent loss of species that can threaten the ecosystem, which is one of the most frightening symptoms of constant biodiversity crisis [8]. Hence, maintaining and/or improving biodiversity is the primary goal of current marine conservation research [9, 10]. Therefore, it prime need of biological studies on PKW to understand the impact of human activities on their health.

Research focusing on understanding the biological events in the body and/or systems of marine mammals has grown in recent years. However, due to sampling restrictions, it is challenging to study the environmental effects on biological processes in marine mammals. However, cells culturing and establishing primary and fibroblast cell lines can provide a unique opportunity for marine conservation research, estimation of mammalian biological responses, underlying molecular mechanisms and indeed animal cloning [9]. Furthermore, cultured cells and cell lines can be used for conservation of genetic resource in the laboratories [11]. Besides, environmental and pathological effects studies on marine mammals are also possible using cell culturing and model development, thus extending to toxicological, bacteriological, virological and epidemiological studies [12]. Considering the critical importance of cell culturing and genetic material preservation in conservation biology laboratories, we focused on establishing a PKW cell line, which will help in broadening research strategies and offer researchers a reliable tool for understanding the biological response and mechanisms of PKW and/or other marine mammals. Importantly, outputs of this study can be valuable in the reprogramming of skin fibroblast into iPSC and specific cell types. In this study, we cultured primary cells from the skin of a PKW and successfully achieved fibroblast cell line PKW-LWHT. The derived fibroblast cells were characterized by morphological observation, immunologic methods and cytogenetical confirmation.

## Materials and methods

### Ethics statement

This animal study (short title: Establishment of cell line) was carried out in strict accordance with the recommendation of the Marine Ethical Committee (Guangdong P.R. China). All experiments were carried out by ethical approval of working guidelines Institute of Marine Biology, Shantou University P.R China with respect to animal experimentation and care of animals under study, and all efforts were made to minimize suffering.

### Collection of sample

A male pygmy killer whale (*Feresa attenuata*) with the body-length of 231 cm and weight of 62 kg was found dead on 24 July 2014 at Longhu sandy beach of Shantou, Guangdong, P. R China. The provincial authorities requested Marine Biology Institute, Shantou University for the postmortem. The whale was found freshly dead within 3–4 hrs. The fluke region was sterilized with soaked (70% alcohol) cotton swabs. The dermal tissue samples with approximately 0.75–01 cm in size were removed aseptically from the fluke close to the marginal line by sterilized sharp scalpel blade and immediately placed into the flask containing medium with Dulbecco’s modified Eagle’s medium (DMEM), Fetal Bovine Serum (FBS) and Antibiotics (Penicillin (200U/ml), Amphotericin B (5μg/ ml) and Streptomycin (200μg/ml).

### Skin sample processing

The skin samples were processed according to Whitworth et al. [13] with slight modifications. In brief, the tissue specimens were washed with Dulbecco’s phosphate buffer saline (PBS, pH-7.2–7.4) and cut into small pieces (approximately1 mm^3^) using sterilized scalpel blade and tweezers. During dissection, epidermis, dermis and blubber were separated. Adipose, vascular, and necrotic tissues were removed carefully. Approximately 12 fragments of skin tissue covering about 0.5 cm^2^ were uniformly distributed in each well of collagen coated 6-well tissue culture plate. To ensure tissue attachment, a sterilized glass coverslip was used to apply slight pressure; culture plates were inverted and then turned over after 20 minutes at room temperature to achieve tight attachment of tissue fragments.

### Cell culture media and growth condition

The attached tissue fragments growth medium was composed of DMEM and Ham’s F12 in an equal (50:50) ratio supplemented with 15% fetal bovine serum (FBS), 0.1 mM non-essential amino acids, 2 mM L-glutamine, and antibiotics (100 U/ml penicillin, 100 μg/ml streptomycin). Attached tissue fragments were incubated in 3 ml medium (pH 7.2), and media was replaced every 48 hrs. The plates were incubated at 37 °C with 5% CO_2_ standard mammalian culture condition and cell growth was monitored at 24 h by an inverted microscope (Olympus, [place of purchase]).

### Primary cell culture and subculture

After 21 days, cell monolayer formation appeared and the tissues were dislodged to a new collagen coated 6 wells plate. The monolayer cells were washed twice with PBS (pH 7.2–7.4). Subsequently, the cells monolayer was detached by trypsin-EDTA (0.25% Invitrogen, USA) using cell digestion technique. After adding trypsin (0.25%), 6 wells plates were incubated at 37 °C for the 90 seconds. The plates were shaken gently and the cold medium with FBS was added to block trypsinization. The detached cells were washed twice with phosphate-buffered saline (PBS, 7.2–7.4), and then centrifuged at 1000 rpm for 10 min. Suspended cells were counted by cytometer and appropriate concentration of seeding was transferred into new 6 wells plate. Subsequent subcultures were maintained at 1:3 (one well of 6 wells plate for three wells of 6 wells plate), when confluency was observed at 85–95% every 7–8 days. The primary cell culture was named as pygmy killer whale Liu Wen Hua (PKW-LWH), and subcultures were continued as the procedure mentioned above every 7–8 days. A part of each cell culture passage was preserved by cryopreservation in liquid nitrogen.

### Karyotype analysis

Cells culture passage 3 primary cells and passage 20 (transfected cells) were prepared for karyotype when a metaphase was developed. For each chromosome preparation, three cultures in 35-mm dishes were used. Colcemid was added to confluent (70–90%) cells at a concentration of 0.6 μg/ml for 14-16h before harvesting for chromosomal analysis. The cells were detached from the culture plate into single cells by trypsinization. To maintain hypotonicity of the cells, the collected cells were treated with 0.075 M KCl for 15 min at room temperature (RT). Following fixation of cells for three times, [missing object] were subjected to freshly prepared Carnoy’s fixative methanol/glacial acetic acid with the ratio of (3:1), for 30 min. The pellets were resuspended in 0.2–0.5 ml of fixative, conditional to cells density the mixed pallets were dropped on cold wet slides. The slides were dried as per standard drying method and before dropping of pellet slides were made cold wet at -20 °C for 1 min. Furthermore, slides were aged at 65 °C for 18h and trypsinized with 0.05%trypsin-EDTA in PBS for 7-10s at 37 °C, and then rinsed twice with PBS. Finally, the slides were stained with Giemsa (10%) in a phosphate buffer (pH 6.8) for 10 min. After rinsed with tap water, slides were dried. The chromosomes were photographed under an inverted microscope.

### Immunofluorescence assay

Fibroblast cells were cultured in bottom glass cell culture dishes until it reached 70–90% coverage. Next, cells were rinsed (PBS) and fixed with pre-chilled 4% paraformaldehyde for 10 min at RT, then washed with PBS and incubated in blocking solution, bovine serum albumin in PBS (1% BSA, Sigma-Aldrich) for 60 mints at rotary shaker. The culture plates were incubated with primary antibody overnight at 4 °C in the wet dark box and diluted in blocking buffer according to manufacturer’s instructions. Primary antibody was removed and the cells were washed with PBS for three times, and the secondary antibody anti-mouse Alexa Fluor 488 (Invitrogen, United States) was then added for 1h at RT. The cells were washed three times in PBS at rotary shaker with each washing for 5 mints and subjected to cell nucleus staining with DAPI for 10 min, and finally embedded in an antifade mounting medium (20 mM Tris-HCl, 0.5% N-propyl gallate, 90% glycerol, pH 8.0) for fluorescence microscopy.

### Polymerase chain reaction and Western blotting

RNAiso (TaKaRa, Japan) was used to extract RNA from fibroblast cells and purified using RNeasy^®^ MinEluteTM (TaKaRa, Japan) according to the manufacturer’s instructions. Total amount of RNA was quantified by Nano Drop (Thermo Scientific). Moreover, RNA was reverse transcribed using Reverse Transcriptase (RTase) M-MLV (RNase H^−^) (TaKaRa). Briefly, 1μg of RNA was combined with 10× first strand buffer, 1.0μl of oligo (dT) primer and RNase-free water up to 10 μl, mixed well and incubated at 70°C for 10 min. Moreover 5×M-MLV Buffer (4μl), 1μ1 of dNTP mix (10mM), RNase Inhibitor 0.5μl (40U/μl), RTase M-MLV 1 μ1 (200 U/μl) and RNase-free water to total volume of 20μl. The mixture was incubated at 42°C for 1h and 30 min; the reaction was stopped by heating at 70°C for 15min, and finally 5min on ice. Oligonucleotide primers for SV-40LT, SV-40ST and Vimentin were designed to evaluate the expressions, based upon sequences available from public databases (Table 1).

**Table 1:** Shows the forward and reverse sequences used in PCR.

Primer name	Forward and Reverse (5’-3’) sequences	BP
Vimentin-F1	GCCGACGCCATCAACACCGAG	229
Vimentin -R1	GCGACTTGCCCTGGCCCTTGAG	
Vimentin -F2	GCAGCTCAAGGGCCAGGGCAA	207
Vimentin -R2	CCTCGACGCGGGCTTTGTCGT	
Vimentin -F3	AGAGAACTTTGCCGTGGAAGCTG	110
Vimentin -R3	TAACATTCAGCAGATCCTGGTATTC	
SV40ST-F	CGAAGCAGTAGCAATCAACC	301
SV40ST-R	CATCCTGATAAAGGAGGAGATG	
SV40LT-F	CATGCTCCTTTAACCCACCT	889
SV40LT-R	GCGACTTGCCCTGGCCCTTGAG	

The cell lysis was performed in lysis buffer (10 mM Tris–HCl pH 7.5, 0.4 M NaCl, 1% NP-40,0.4% Triton X-100, 0.2% sodium deoxycholate, 1 mM EDTA, protease inhibitors) on ice for 30 min. Then cells lysate was centrifuged at 12,000 rpm for 10 min. The supernatants were collected and boiled in 5× SDS loading buffer, and separation was achieved by sodium dodecyl sulfate-polyacrylamide gel electrophoresis (12% Tris-HCl gel). The proteins were separated by gel transferred onto a nitrocellulose membrane. The membrane blocking was performed by 5% nonfat milk in TBS with Tween-20 (TBST; 20 mM Tris-HCl pH 7.6, 150 mM NaCl, 0.05%Tween-20) at room temperature (RT) for 1 h, and then was incubated overnight with primary antibody (dilution as per instructions) at 4°C with gentle shaking. Following three washes in TBST for 10 min each, the membrane was incubated with secondary antibody (alkaline phosphatase-conjugated anti-mouse IgG antibody, Sigma) for 1 hat RT with gentle shaking. The membrane was washed three times with TTBS for 5 min and then reacted with luminescent chromophore before being exposed to film for less than 1 min.

### Fibroblast cells transfection

To achieve transfection, Lipofectamine 2000 reagent (Invitrogen) was used per manufacturer instructions. We used plasmids to transfect the dermal cell cultures of the PKW. The plasmid pSV40LT-Puroen- codes the Simian vacuolating virus 40 (SV40) large T- antigen. In addition, cytoplasmic green fluorescent protein (GFP) expressing pEGFP-N3, and pEGFP-C1 were induced to observe the transfection rates. To obtain stable cell clones expressing the SV40 large T-antigen, at day-2 after transfection, cells were exposed to 200 μg/ml of geneticin (G418, Thermofisher) in a complete medium. After another 3 days, the dead cells were removed, and the surviving cells were reseeded at 105 cells/10-cm dishes with complete medium containing 200 μg/ml of geneticin for clonal growth. The geneticin resistant cell colonies were picked for expansion and subsequently examined for the SV40 large T-antigen expression by RT- PCR, Western blot and immunofluorescence.

### Cryopreservation and recovery

Approximately 1×10^6^ of PKW-LWH cells (Passage-3, 7 and 10) were used for cryopreservation. Cells were washed with 5 ml of 1 × DPBS, trypsinized with 1 ml 0.25% trypsin and centrifuged at 1000 rpm for 10 min. The pellet was resuspended in freezing medium containing 90% FBS and 10% dimethyl sulfoxide (DMSO). Cryotubes were frozen overnight to -80 °C using an isopropyl alcohol freezing container and final storage was conducted in liquid nitrogen. For recovery, frozen cells were rapidly thawed to room temperature and resuspended in the pre-warmed medium. Before plating in cell culture flasks, survival was measured (passage-3) by using the Trypan blue exclusion assay.

### Growth curve

Transfected PKW-LWHT and untransfected PKW-LWH cells were cultured to evaluate the continuous growth rate according to [14]. In brief, single cells were evenly seeded into 24-well plates at 1×104 cells per well to analyze growth for 1 to 10 days and the ½ medium was replaced with fresh medium/24h. Cells were harvested at 24h interval up to 10 days. A final volume of 1 ml brought up to for cell number count by hemocytometer. Trypan blue stained cells were considered as dead cells and non-stained cells were counted. Triplicate copies of cells were counted each time, and the mean cell counts were used to construct the growth curve.

### Data analysis

Experiments were carried out in triplicate for each analysis. Statistical analysis was performed using SPSS Version 16. The results were presented as mean ± S.D. Differences in the PKW-LWH and PKW-LWHT growth rate were analyzed with a one-way analysis of variance (ANOVA).

## Results

### Primary cell culture and subcultures

**Fig 1:**
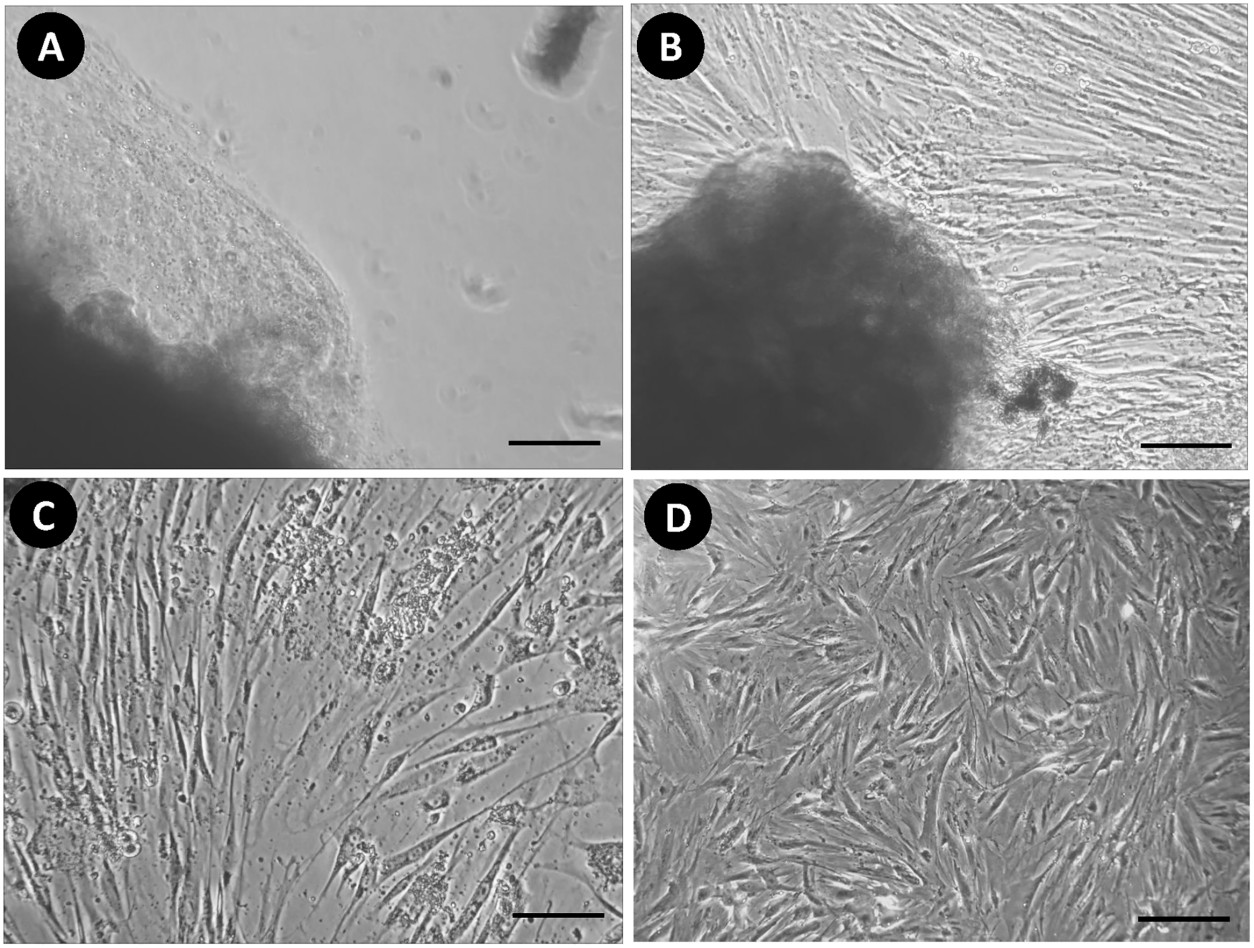
Phase contrast appearance of PKW-LWH primary cells. (A) Tissue attachment at day first to the collagen coated plate, (B) primary cells growth from connective tissue fragments, (C) elongated spindle shaped morphology of fibroblast cells, (C) isolated primary cells with confluent layer observed at day 7 in the six well plate. The scale bar indicates 100μm.

**Fig 2:**
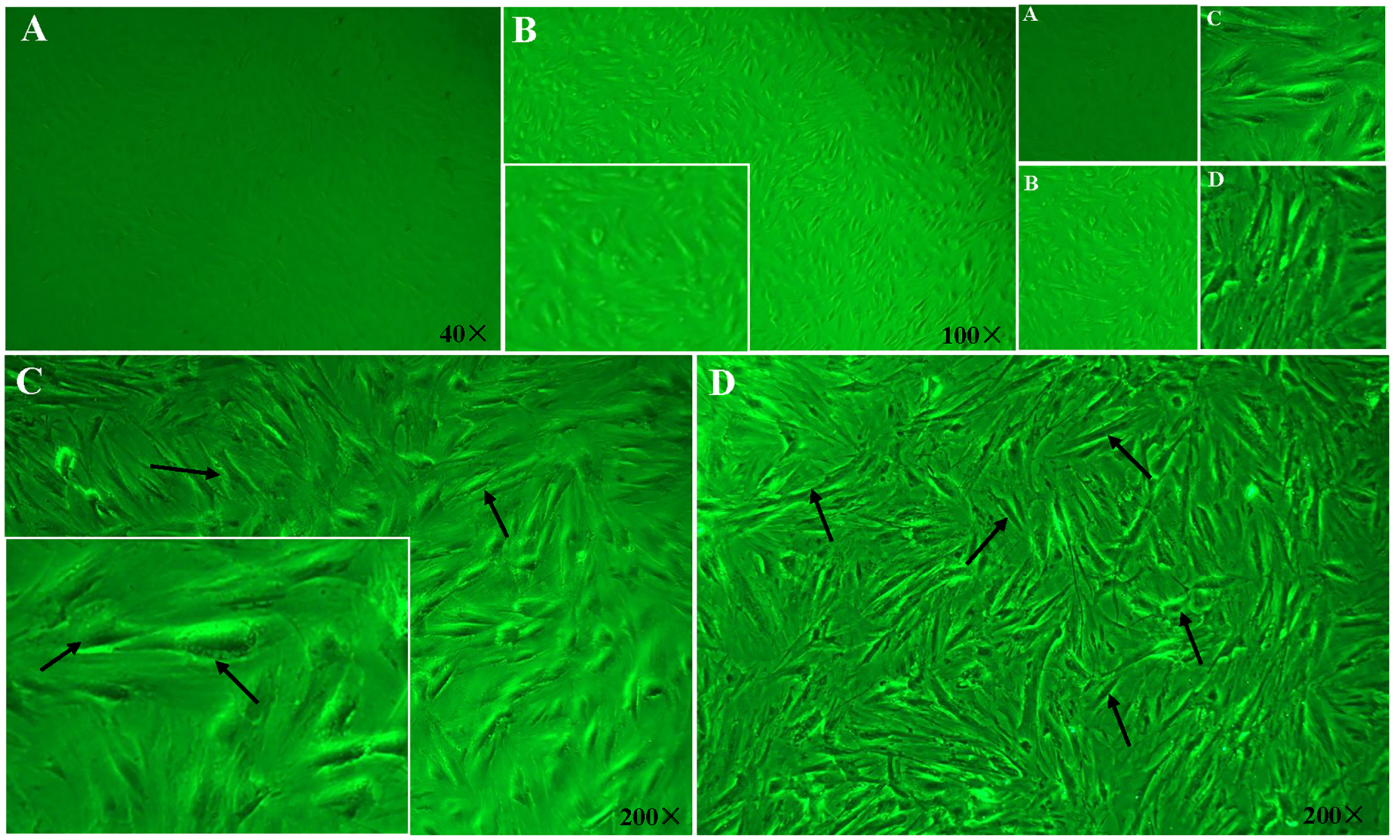
Morphological description of PKW-LWH. A, B Primary pure culture of a Pygmy killer whale skin free of lipid droplets (40x and100x), (C) passage two cells showing spindle shaped morphology at 200x indicated by black arrows, (D) passage three cell culture, showing various stages of cells growth short, long and elongated in shape.

Dermal fresh samples of a PKW were obtained and five independent primary cultures were processed. The primary culture appeared after 16 days of tissues attachment. A few single cells growth was observed at the edges of attached tissue and morphologically the cells showed adherence to the plate. On the 22^nd^ day of primary culturing, morphologically fibroblastic cells with polygonal and spindle-like shaped appeared in the culture plates and were photographed under phase contrast microscope (Fig 1). Attached tissues were detached from the plate carefully and the monolayer was used to achieve the first subculture (1:3). The passage-1 growth density was noted at 80–90% within 7–8 days and named PKW-LWH. Meanwhile, various stages of cell growth were observed morphologically and prominent spindle shaped cells appeared in passage-2 (Fig 2). A total of 13 passages of primary culture was achieved, the partial growth arrest and morphological changes were noted.

### Karyotype

**Fig 3:**
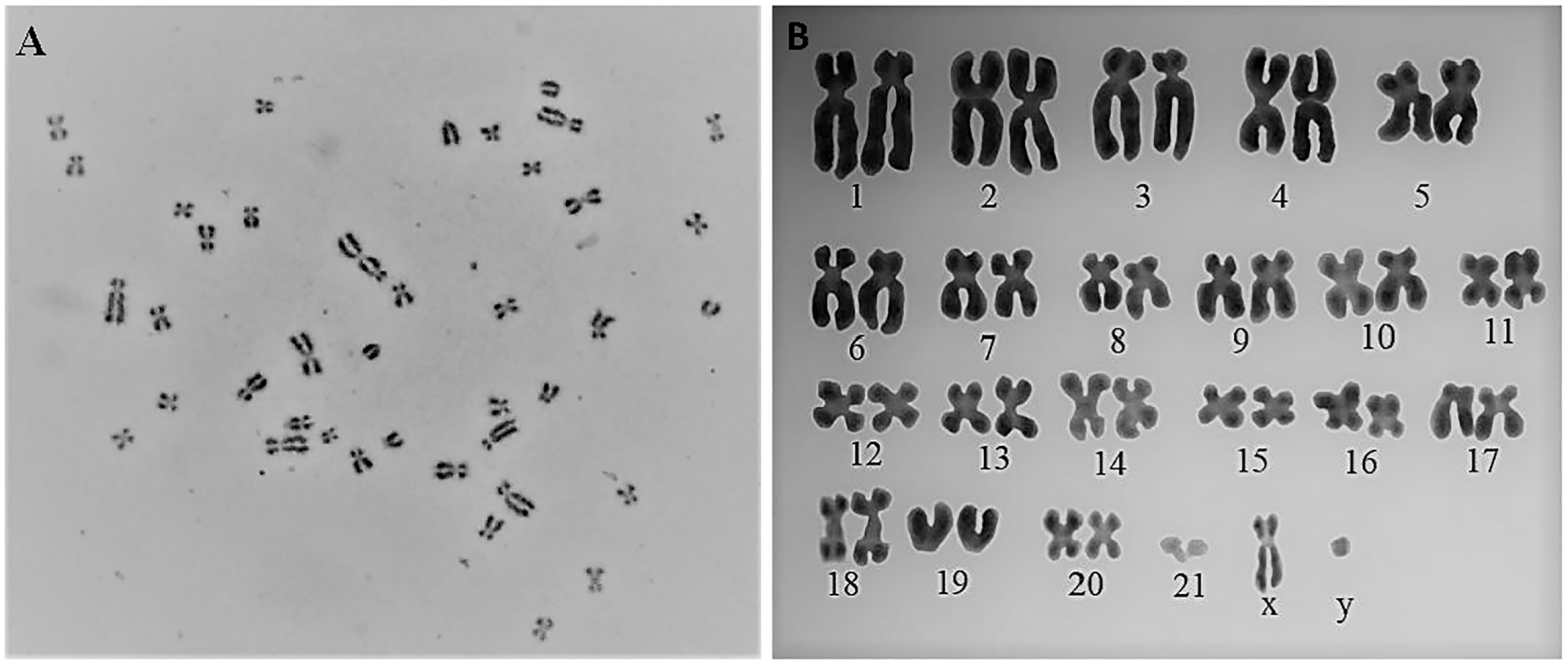
Karyogram of pygmy killer whale cell cultures. (A) PKW-LWH (P3) show chromosome numbers 2n = 44 and male gender. (B) PKW-LWH morphological observation manifested metacentric, sub metacentric and telocentric stages of chromosomes.

The PKW-LWH skin fibroblast cells passage-3 (primary cells) and passage 20 (transfected cells) were prepared for the karyotype. Cell metaphases stage was used to determine the karyotype. The average cells exhibited diploid chromosome pairings with 2n = 44 including 21 pairs of autosomes and 1 pair of sex chromosomes (X and Y). It was observed the PKW-LWH cells were of male origin and the morphological observation showed metacentric, submetacentric and telocentric stages of chromosomes (Fig 3). While, fibroblast cell line karyotyping showed tetraploid chromosomes, however chromosomal aberration was not observed (S1 Fig).

### Identification of dermal fibroblast

**Fig 4:**
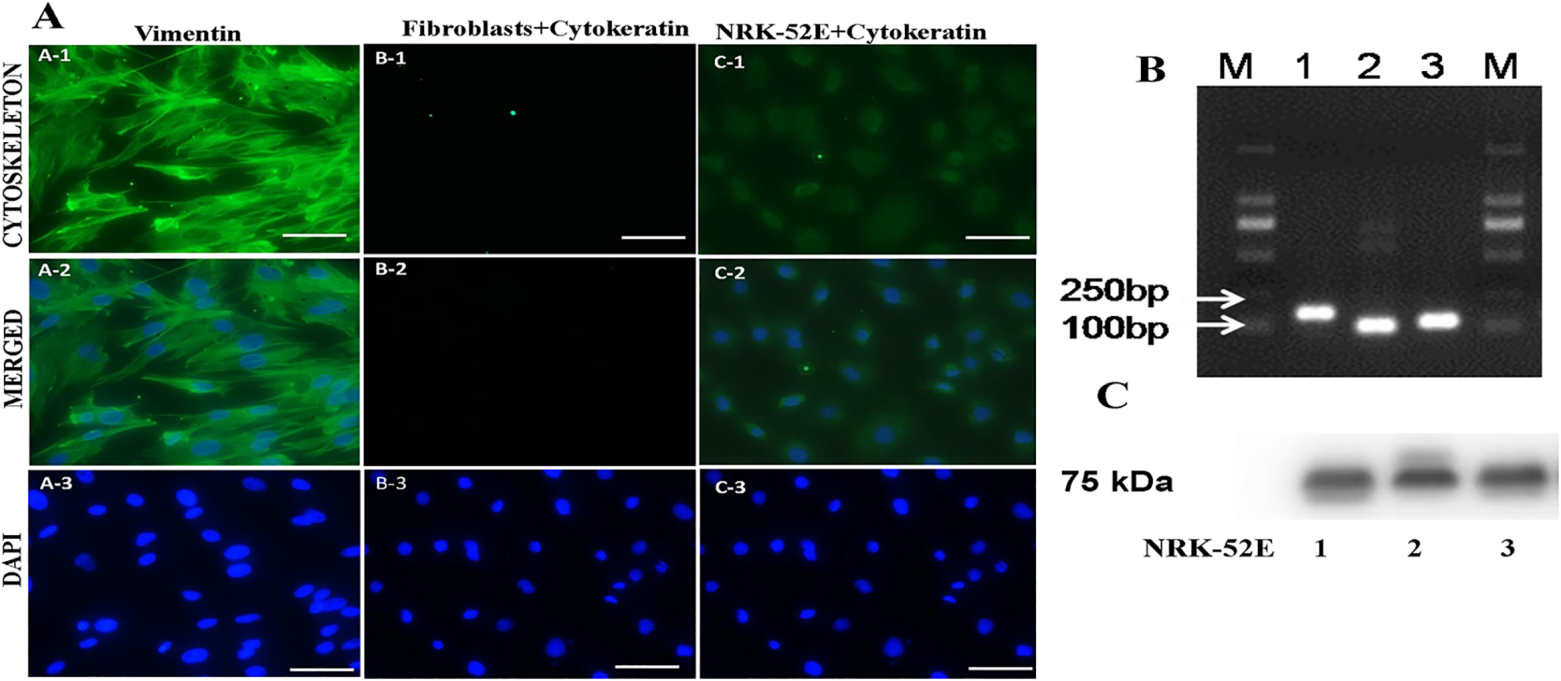
Identification of fibroblast by specific protein marker, RT-PCR and Western blot in PKW-LWH cells. A, Cells exhibited positive expression for intracellular vimentin, merged with DAPI and DAPI showed prominent nucleus (A-1, A-2 & A-3 respectively). No reactivity of cytokeratin with fibroblast (B-1, B-2), and nucleus with DAPI (B-3). NRK-52E, exhibited immunoreactivity with cytokeratin bright green cytoskeleton (C-1) merged cell with DAPI (C-2), and blue prominent nucleus (C-3). B, vimentin expression by RT-PCR and (1,2,3) pair of primers were used. C, western blotting expression (1,2,3) indicates Passage-3, 5 and 10 respectively. The scale bar indicates 50μ m.

Vimentin is an intermediate filament that can indicate the mesenchymal origin of fibroblast cells. We used vimentin as dermal fibroblast marker and found strong immunoreactivity of PKW-LWH to vimentin (Fig 4A). However, cytokeratin (epithelium cells marker) were found to be non-immunoreactive, whereas technical control (NRK-52E cells) exhibited immunoreactivity to cytokeratin antibodies. Further, RT-PCR was used to confirm the expression of vimentin gene in the cultured fibroblast cells and results showed vimentin expression (Fig 4B). The western blotting manifested vimentin protein expression, which appeared at about 94kD (Fig 4C). Therefore, these results lend strong support that spindle-shaped cells PKW-LWH1 cells were indeed fibroblasts.

### Recovery after cryopreservation

**Fig 5:**
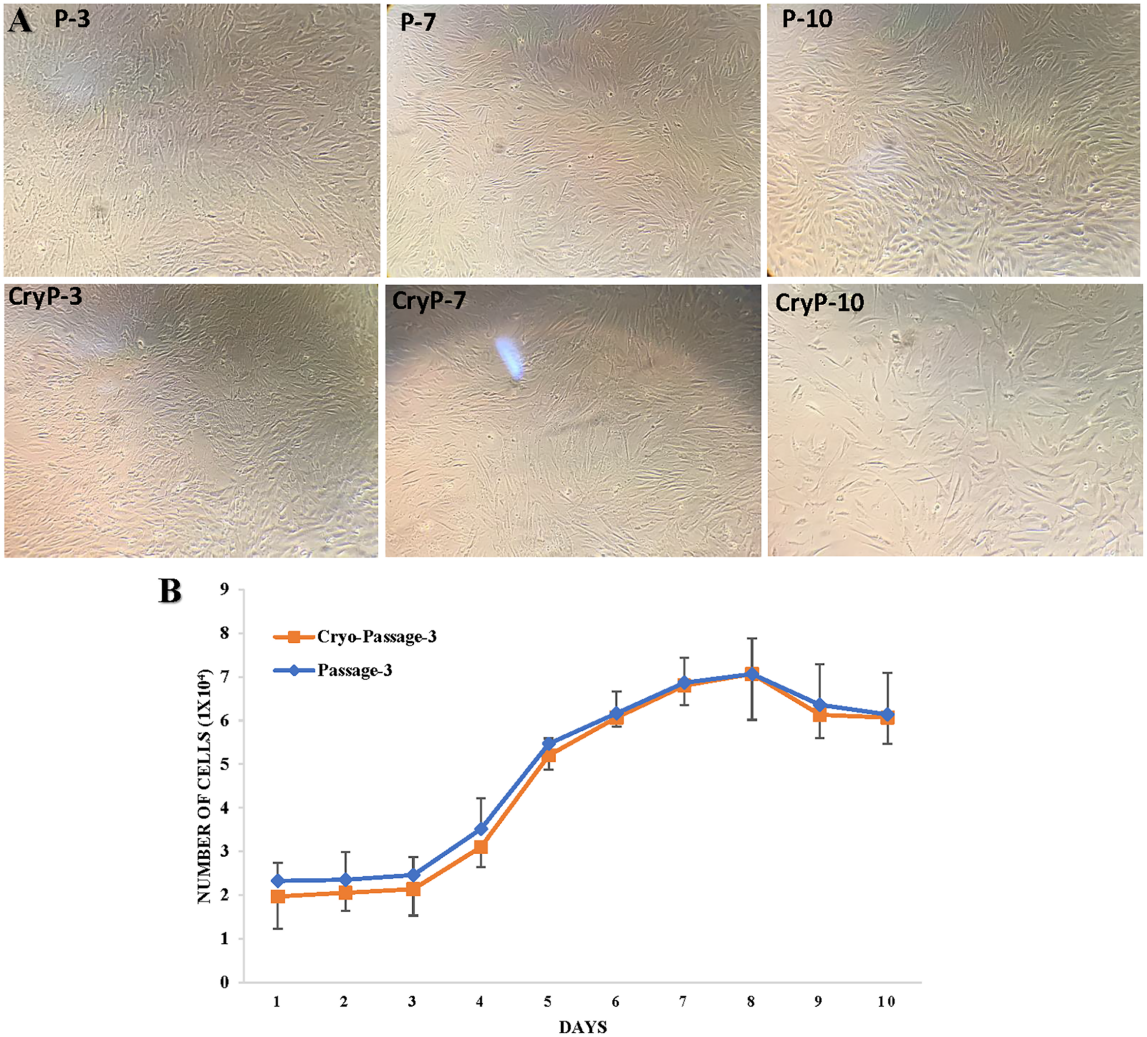
Morphology and growth curves of PKW-LWH cryopreserved and transferred passage. Passage-3, 7 and 10 are shown with cryopreserved passages (CryP-3, 7 and 10), shows similar morphology. Growth curve comparison of (P-3 and CryP-3) indicate similar growth properties and after thawing slightly reduced growth first 4–5 days in cryopreserved (CryP-3) cells. Data represent the mean of three technical replicates and the error bars represent the SD.

Cryopreservation is often employed to enable the long-term back-up of cells because it minimizes morphological changes, genetical variation and contamination of preserved cells. Results showed that PKW-LWH cells passage-3, 7 and 10 were successfully revived to establish the preservation recovery growth ability (Fig 5A). Viability was assessed by trypan blue staining, and it showed ~ 95% cells were alive. Meanwhile, the growth of cryopreserved and subculture cells passage-3 comparison showed a similar growth curve suggesting [missing information] (Fig 5B).

### Transfection efficiency and G418 resistance cells

**Fig 6:**
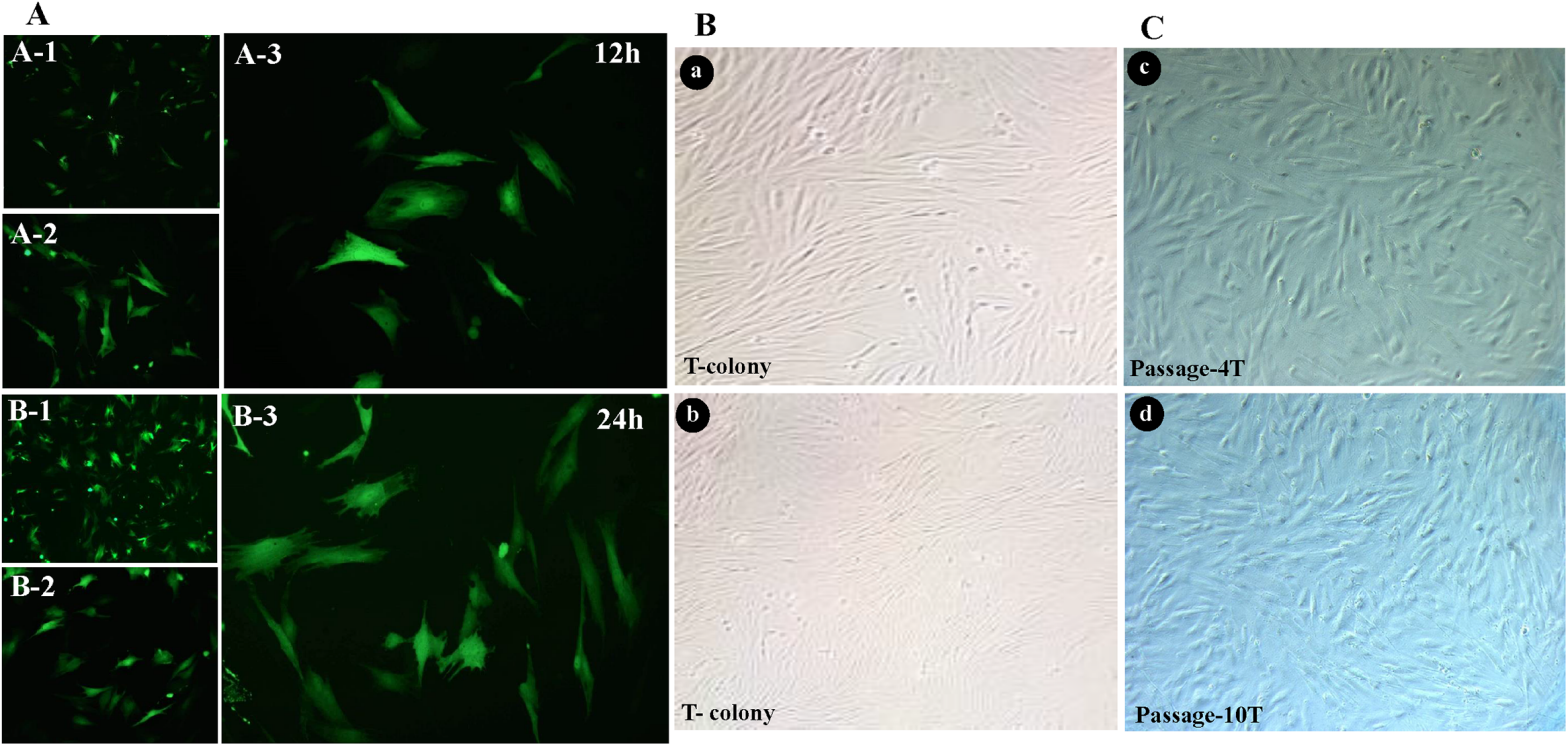
Pygmy killer whale primary cell lines PKW-LWH transfection rate. A transfection was observed under immunofluorescence microscope. Transfection rate at 12h (A-1, 2 and 3), after 24h (B-1, 2 and 3). B, colony growth of transfected cells (a,b) and C, the morphology of transfected passage 4 and 10 (c, d).

The PKW-LWH were transfected with plasmids pEGFP-N3, and green fluorescence protein (GFP). The transfection was observed under immunofluorescence microscope (Fig 6A) and detected using GFP intensity in PKW-LWH cells. It was found ~70% and ~90% GFP positive for 12 h and 24 h post transfection respectively. Neomycin G418, the concentration was optimized 200 μg/mL (data not shown) and added in the medium and the non-transfected cells were dead in 5 days while transfected cells remained while continuing to grow. Positive G418-resistant colonies of rapidly proliferating morphologically were screened and proliferated into new plates (Fig 6B). Meanwhile, transfected cells P-5 and P-10 were cultured and there was no morphological difference and growth variation after cryopreservation was observed (Fig 6C).

### Transfection confirmation and post-transfection cells growth

**Fig 7:**
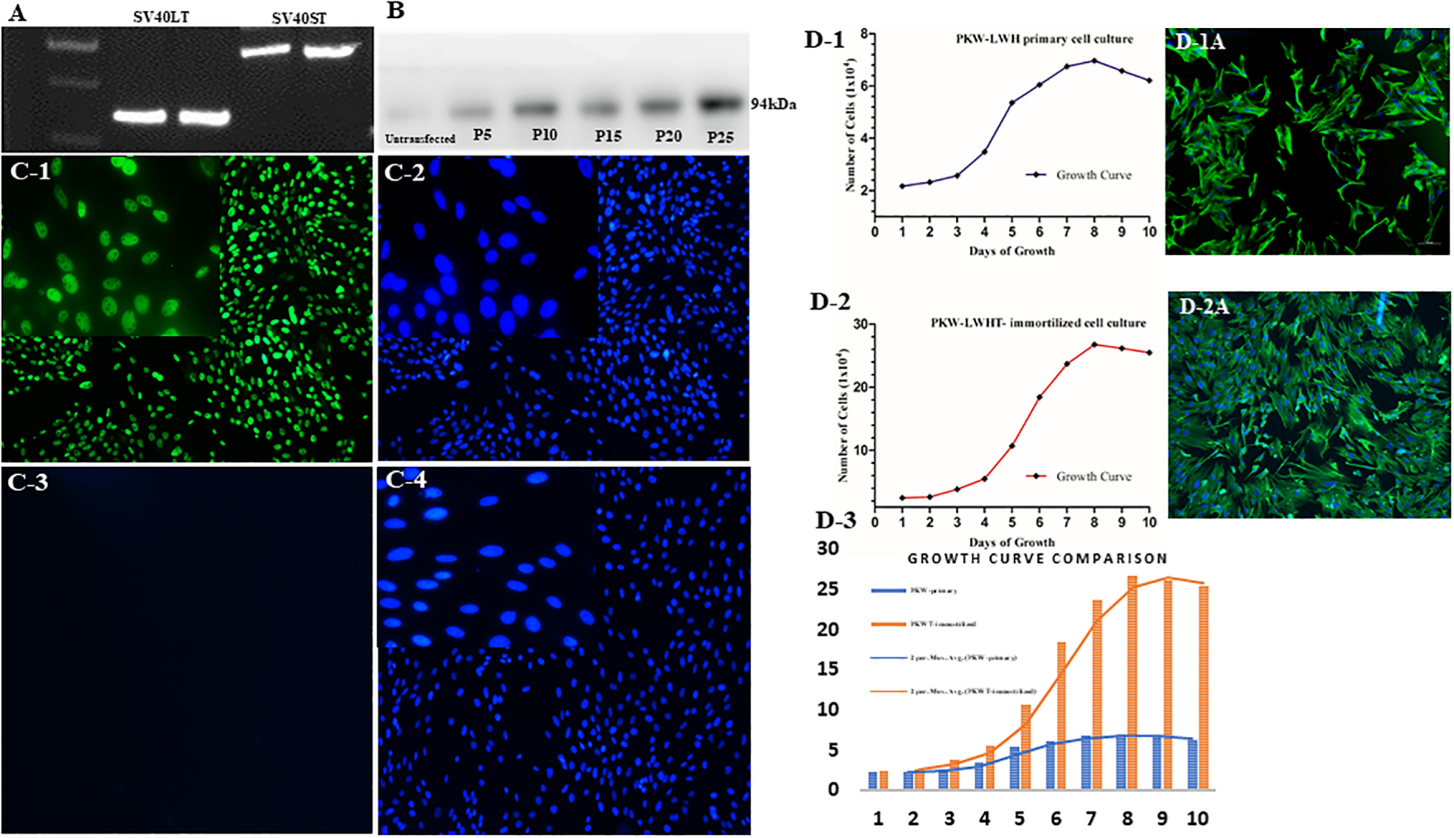
Transfection confirmation of PKW-LWHT cells. A, shows RT-PCR amplify two bands reveals the expression of SV40LT and SV40ST genes in PKW-LWHT. B shows SV40 virus large T protein (passages, 5,10,15,20 and 25) by western blot indicates stability of transfection. Immunofluorescence C-1 shows SV40LT location (green), C-2 only nucleus (blue). PKW-LWH, (C-3) did not show SV40LT immunoreactivity and C-4 shows nucleus of untransfected cells. D, shows growth curve of primary cells (D-1), growth curve of transfected cells (D-2) and comparison of primary and transfected cells (D-3), the phenotype morphology of primary cell and transfected cells presented (D-1A and D-2A) respectively.

Simian virus 40 early regions (SV40 ER) proteins, LT and ST, play key roles in transfection and transformation. RT-PCR and western blot were employed to detect functional gene of SV40 large T and small T in the fibroblast cell line PKW-LWHT. RT-PCR showed that PKW-LWHTcells could amplify two bands revealing the expression of SV40LT and SV40ST genes, and further confirmation was achieved by sequencing (Data not were shown). The negative control group PKW-LWH did not show SV40LT and SV40ST gene bands (Fig 7A). Western blot results indicated that SV40 Virus Large T protein was stably expressed in transfected cells with a size of 94 kDa (Fig 7B). Whereas, the negative control PKW-LWH, untransfected cells did not manifest protein expression. Furthermore, immunofluorescence expression was determined and green nucleus with distinct intensity appeared in PKW-LWHT cells (Fig 7C-1) and remained absent in untransfected cells (Fig 7C-2).

Whereas, non-transfected cells (negative control) PKW-LWH cells (Fig 7C-3) did not show green fluorescence although DAPI manifested clear outline of cell nuclei (Fig 7C-4). Viewed under a microscope, the cultured skin cells exhibited a similar morphology of fibroblast before and after transfecion.

Furthermore, the growth curve showed (Fig 7D) that untransfected PKW-LWH cells at (passage- 4) reached the highest growth on the 8^th^ day of culture, and cell doubling time was 68.9 h. In comparison, the fibroblast PKW-LWHT cells reached the highest growth on the same day (8^th^ day), but the cell doubling time was faster at only14.41 h. Furthermore, the growth curve of PKW-LWHT cells was markedly higher at 4^th^ day to 8^th^ day of culture than the PKW-LWH cells. However, phenotype morphology of primary cells and transfected cells (cell line) remained unchanged (Fig 7D).

## Discussion

In the present study, we developed dermal fibroblast cell line (PKW-LWH) and established fibroblast cell line (PKW-LWHT1) by transfecting SV40 T antigen gene into a primary cell line. A similar approach was applied to develop primary cell culture line from humpback whale skin tissue [15] and to establish the fibroblast cell line from Chinese white dolphin [16], and skin fibroblast from another cetacean species, Yangtze finless porpoise [11].

Initially, the PKW skin tissue was attached to culture plates, and edges appeared few single cells growth around it after 16 days of tissues attachment. Cells morphologically manifested adherence with culture plate. The primary cultures appeared morphologically fibroblastic cells with spindle-like shape and squamous. Thirteen passages showed similar growth patterns but passage 14 showed prominent morphological changes (mega/giant cells structure) and cells growth achieved in 12–14 days. Whereas, findings of Burkard et al. [15] previously described that established epidermal cell cultures exhibit squamous cells morphology, express vimentin, and grow very well until passage thirty. The growth rate of the primary cells culture with higher passages slows down, presumably as a result of senescence [16]. To overcome cell culture morphological modulation and growth arrest, SV40 transfection induces forced expression of large T-antigen, leading to the production of cell lines and enabling them capable of continuous proliferation [17].

Karyotype analysis of dermal fibroblast cells of the pygmy killer whale (primary culture) revealed normal male karyotype with 22 pairs of chromones. Moreover, it was noted that after multiple passages, all karyotypes from PKW-LWH epidermal cultures were apparently manifested euploid. It was previously reported that the normal diploid somatic cells of cetacean species contain 21 or 22 pairs of chromosomes [8], which can show multiple stages of chromosomes. Similarly, Jin et al. [16] showed 44 chromosomes in Chinese white dolphin fibroblast cells. Cetacean chromosomes numbers often have 42 to 44 and may appear metacentric, submetacentric, telocentric and sub-telocentric [11]. Thus, these cell cultures may be useful for assessing genotoxic agents and for chromosomal breakage analysis.

Vimentin is considered as an intermediate filament indicating the mesenchymal origin of endothelial and fibroblast cells. Previously, vimentin as a marker was widely used to identify the fibroblast cells [18]. Vimentin has shown to be positively expressed in fibroblast cell of other cetacean species [19, 20]. Here, we also used vimentin as dermal fibroblast marker and found strong immunoreactivity to vimentin. No immunoreactivity was observed by cytokeratin, but positive control NRK-52E had strong immunoreactivity with cytokeratin (epithelial identification protein marker). Further, RT-PCR was used to confirm the vimentin gene expression and western blotting revealed vimentin protein. Thus, altogether immunoreactivity, RT- PCR western blotting and morphologically spindle-shaped cells lend strong support that PKW-LWH were indeed fibroblasts.

The preservation recovery ability of PKW-LWH cells after cryopreservation was assessed and results showed ~ 90% cells were trypan blue positive and growing well. Meanwhile, the growth of cryopreserved cells was similar to the subculture and growth curve was also noted parallel. Another study showed that cryopreservation was applied from early passages and revival manifested 90% cells recovery after cryopreservation [11]. The cryopreservation method by Stacey and Masters [21], demonstrated that long-term backup of cells and/or genetical material is required to protect the cells from morphological changes, genetical variation, and possible contaminations.

Primary cells lifespan is limited to several passages and limits the long-term availability of these cells for further research. Similarly, Jin et al., [16] found that fibroblast cell cultures morphology and growth noted rapid at early passages, but later showed obvious growth arrest at higher passages. To overwhelm this issue, gene transfer of SV40 early regions has been widely used to escape the Hayflick limit [17, 22]. Cell transfection and transformation is achieved by Simian virus 40 early region (SV40 ER) proteins, LT and ST [23]. PKW-LWH were transfected with plasmids pEGFP-N3, and green fluorescence protein (GFP) was used as a marker for positive clones. The transfection was observed under immunofluorescence microscope. The transfection rate appeared very successful after 12h and 24h with ~70% and ~90% GFP positive cells, respectively. However, two different transfection methods plasmid and lentivirus showed similar transfection rate in mammalian fibroblasts [16].

Functional ability of SV40 large T and small T were determined by RT-PCR and western blotting. The common target gene for cell line is SV40 gene, which infects cells with SV40 virus or SV40 gene can be used to convert normal cells into cell lines in vitro [22, 24]. SV40 infected cells genome can be carried out in the nucleus transcription and replication [25]. T antigen plays a critical role in cell transformation and expresses continuously to maintain the phenotype of transformed cells. T antigen enhances cell transformation and maintains the transformed cell phenotype with T antigen [26].

Recently reported that fibroblast cells successfully reprogrammed into neuronal cells by single and multiple factors, [27, 28]. Several studies successfully converted the mammalian (human and animals) fibroblast cells into cardiomyocytes, hepatocytes and pancreatic cells and so on [29–30]. Besides, dermal fibroblasts are used as a neurodegenerative disease marker too [31]. These evidences indicate the effectiveness of exogenous gene transfer into marine mammal’s fibroblast cells, which encourages the researchers to establish multiple cell lines and an inducible pluripotent stem cell that entirely will open the new research directions to explore unveiled finding of marine conservation biology.

## Conclusions

In conclusion, this is the first study that reports establishment of pygmy killer whale fibroblast cell line. Findings provide unique opportunity to develop advancement in integrated research of toxicological and pathological effects on marine mammals. Future research will be extended to cell reprogramming by epigenetically-based cell conversion to obtain induced pluripotent stem cells and/or conversion of somatic cells into a specific type of cells.

## Supporting information

S1 Fig Figure shows chromosomes post transfection (passage 20), tetraploid numbers were observed and no chromosomal aberration (abnormally) manifested, figure represents the replication results. (JPG)
