# Supplementary figures and images for: Establishment and characterization of pygmy killer whale (Feresa attenuata) dermal fibroblast cell line
Source: PLoS One. 2018 Mar 29;13(3):e0195128. doi: 10.1371/journal.pone.0195128 (PMC5875847; doi:10.1371/journal.pone.0195128)

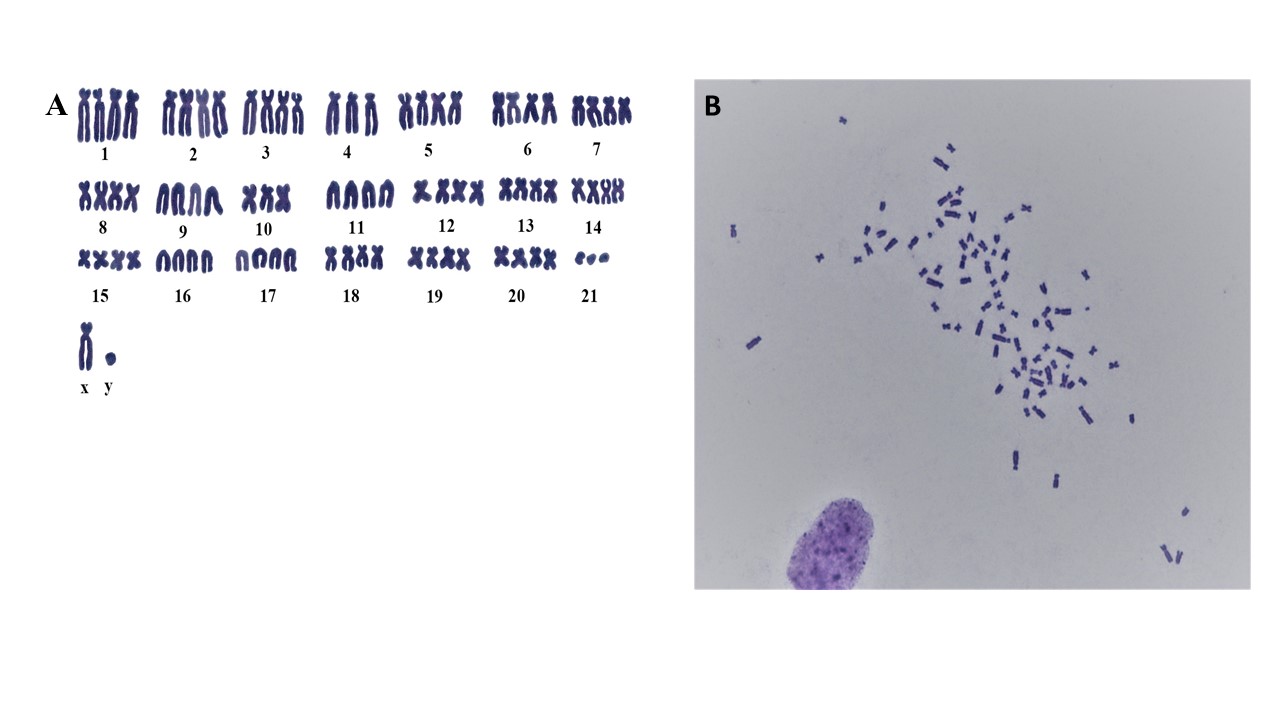

Supplement: S1 Fig — (JPG) [file pone.0195128.s001.jpg]
